# Optimization of Electrode and Cell Design for Ultrafast-Charging Lithium-Ion Batteries Based on Molybdenum Niobium Oxide Anodes

**DOI:** 10.1021/acsaem.2c01814

**Published:** 2022-08-12

**Authors:** Yazid Lakhdar, Harry Geary, Maurits Houck, Dominika Gastol, Alexander S. Groombridge, Peter R. Slater, Emma Kendrick

**Affiliations:** † School of Metallurgy and Materials, University of Birmingham, Edgbaston, Birmingham B15 2TT, U.K.; ‡ Echion Technologies Ltd., 9 Cambridge South, West Way, Sawston, Cambridge CB22 3FG, U.K.; § School of Chemistry, University of Birmingham, Edgbaston, Birmingham B15 2TT, U.K.

**Keywords:** niobium oxide, lithium-ion batteries, rate, fast charging, electrode density, cell balancing

## Abstract

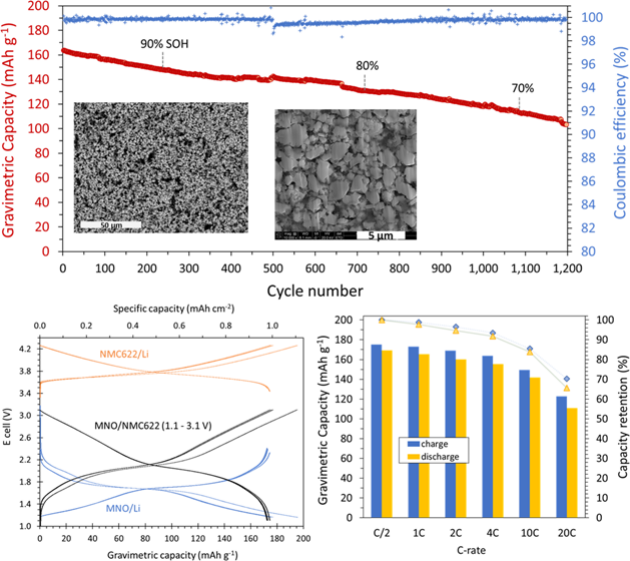

Niobium oxides are an emerging class of anode materials for use in high-power lithium-ion batteries. Galvanostatic cycling and electrochemical impedance spectroscopy (EIS) were used in this study to investigate the influence of electrode porosity, electrode mass ratio, and cycling rate on the capacity, cycle life, and ionic conductivity of Li-ion battery cells based on a modified micron-sized MoNb_12_O_33_ (MNO) anode powder. Both electrode and cell designs were found to have a significant impact on the rate performance and cycle life of Li-ion half- and full cells. A higher specific capacity, improved rate performance, and a longer cycle life were obtained in both anode and cathode half-cells by lowering the electrode porosity through calendaring. MNO/Li half-coin cells displayed excellent cyclability, reaching 80% state of health (SOH) after 600 cycles at C/2 charge and 1C discharge. MNO/NMC622 full-coin cells displayed a high capacity of 179 mAh g^–1^ at 100 mA g^–1^ (0.5 mA cm^–2^) and excellent cyclability at 25 °C, reaching 70% SOH after over 1000 cycles at 1 mA cm^–2^ after optimizing their N/P ratio. Excellent cyclability was obtained at both 1C/1C and fast 2C/2C cycling, reaching 80% SOH after 700 and 470 cycles, respectively. Full-coin and small pouch cells had outstanding rate performance as they could be charged from 0 to 84% capacity in less than 5 min at 10 mA cm^–2^ and to 70% SOC in 120 s at 20 mA cm^–2^.

## 1 Introduction

Lithium-ion batteries (LIBs) are an essential technological enabler of our modern society, powering our smartphones, laptops, power tools, and electric cars, and LIBs are becoming ever more important as a grid-scale storage of renewables and the electrification of vehicles is gaining significant traction globally.^1,2^ Electric vehicles (EVs) require rechargeable batteries that (i) are safe, (ii) can store more energy for increased range, (iii) deliver more power for faster charging times, and (iv) can go through more charge–discharge cycles for a longer operating lifetime.

Commercial LIBs currently used in EVs are based on cell chemistries with an intercalation-type graphite anode against various possible layered transition-metal oxide cathodes such as LCO, LMO, NMC, NCA, or LFP.^3−5^ Although graphite has a high theoretical specific capacity of 372 mAh g^–1^ and good cycling life, it also has some inherent limitations. Indeed, the growth of a thick solid-electrolyte interface (SEI) layer formed on the surface of graphite anodes results in increased impedance and irreversible consumption of a large amount of Li^+^ ions.^6,7^ Moreover, the low electrochemical potential for lithium intercalation in graphite (<0.1 V vs Li/Li^+^) leads to lithium dendrite formation during fast charging, which limit the cycle life of graphite-based LIBs and raises serious safety concerns.^8,9^

Several alternative anode materials have been investigated and implemented to improve the cycle life, safety, and rate performance of LIBs.^10^ Lithium metal for solid-state batteries is highly promising due to its ultrahigh specific capacity (3860 mAh g^–1^), but significant improvements still need to be made to mitigate Li-dendrite growth.^11^ Similarly, pure silicon and Si oxides (SiO*_x_*) have been investigated extensively as the theoretical specific capacity of Si-based anodes is 1 order of magnitude higher than that of graphite. However, the practical implementation of Si-based anodes for commercial applications is limited by the dramatic volume expansion of 100–300% experienced by these materials upon cycling, which results in extensive particle cracking, low initial Coulombic efficiency, and poor cycle life.^12,13^ Therefore, the use of silicon/graphite composite anodes with 0–20 wt % of Si nanoparticles has been favored, although it tends to increase both manufacturing complexity and cost.^14−16^

Li_4_Ti_5_O_12_ (LTO) has a high intercalation potential (1.55 V vs Li/Li^+^) resulting from Ti^4+^/Ti^3+^ redox couple that makes it inherently safer than graphite by eliminating dendrite formation but results in lower voltage and hence lower energy density in a full cell.^17^ LTO also has excellent cyclic stability and has been widely demonstrated as a good anode material candidate for high-rate applications.^18^ However, the low theoretical capacity of LTO (175 mAh g^–1^) limits its suitability for high energy and high voltage applications. Moreover, it needs to be nanosized and/or undergo surface modification^19^ or doping^20^ for high charge/discharge capability due to its low Li-ion diffusion coefficient, which results in increased cost and severe limitations on achieving high electrode volumetric capacities (i.e., >300 mAh cm^–3^) and volumetric energy densities (i.e., >200 Wh L^–1^). This work investigated a material that belongs to a nascent class of intercalation-type anodes: Wadsley–Roth crystallographic shear structure niobium oxides, which much like LTO have a dominant Li^+^ intercalation potential above 1.0 V (vs Li^+^/Li). Furthermore, niobium oxides have a higher theoretical specific capacity than LTO and a theoretical volumetric capacity up to three times as high as LTO,^21^ which could significantly increase the energy density of high-power LIBs.

Wadsley–Roth phases based on niobium oxide were first reported for use in lithium-ion batteries by Cava et al. in 1983.^22^ Since TiNb_2_O_7_ was proposed as an alternative anode by Goodenough’s group in 2011,^23^ a large number of Wadsley–Roth niobium oxide materials were developed and demonstrated in recent years, including Ti_2_Nb_2*x*_O_4+5*x*_ (where *x* = 2, 5, 24),^24^ Al_0.5_Nb_24.5_O_62_,^25^ PNb_9_O_25_,^26^ WNb_12_O_33_,^27^ and MoNb_12_O_33_,^28^ GeNb_18_O_47_,^29^ Cr_0.5_Nb_24.5_O_62_,^30^ AlNb_11_O_29_,^31^ and FeNb_11_O_29_.^32^

Wadsley–Roth crystallographic phases are characterized by open ReO_3_-like channels that enable high capacity and fast Li^+^ diffusivity, combined with crystallographic shear planes that enable excellent stability and reversibility.^33^ The double Nb^5+^/Nb^4+^ and Nb^4+^/Nb^3+^ redox couple of niobates also contributes to a high theoretical capacity, alongside the potential to include other redox-active cations.

In this work, we investigated the electrochemical performance of a modified Wadsley–Roth MoNb_12_O_33_ molybdenum niobium oxide (MNO) powder provided by Echion Technologies Ltd., which has developed and patented a range of mixed niobium oxide materials for high-power Li-ion cells.^34^

MoNb_12_O_33_ was first proposed by Zhu et al. in 2019 in the form of porous microspheres synthesized through a one-step solvothermal method followed by low-temperature calcination.^28^ Their MNO/Li half-coin cell had a high specific capacity (∼320 mAh g^–1^) with a minimum voltage cutoff of 0.8 V, excellent rate capability with a capacity retention of 73% at 10C relative to 1C, and exceptional cyclability at both 1C and 10C. These properties were retained in an MNO/LiMn_2_O_4_ full-coin cell. However, they used extremely thin electrodes with a mass loading of only 1.0 mg cm^–2^, while the weight ratio of conductive carbon black (25 wt %) in both the anode and cathode electrodes was significantly higher than typically used in commercial battery electrodes, which can lead to misleading results.^35^ Furthermore, only coin cells assembled in a glovebox under argon were tested, and no information was given either on the volume of electrolyte used or on the porosity of their electrodes. Finally, compared to the literature on similar materials, a low minimum voltage cutoff of 0.8 V was used, which increases the experimental capacity but is typically a disadvantage in full-cell applications. Indeed, the wider the voltage window for practical use, the more complex, costly, and heavy power electronics and pack-level modifications are required.

Here, we proposed to evaluate the electrochemical performance of MNO anodes with a 90:5:5 ratio by weight of active material, conductive carbons, and PVDF, respectively, used in coin and pouch cells assembled in a dry room environment to evaluate and demonstrate the applicability of this anode toward commercialization. These cells were designed for power applications, and therefore an areal capacity of ∼1 mAh cm^–2^ was targeted, which was significantly higher than most previously published work on similar niobate anode materials in the literature.^28,36,37^ A capacity of 1–2 mAh cm^–2^ is commercially relevant for high-power applications, such as an A123 M1A high-power battery, which was found to have an anode areal capacity of ∼1.25 mAh cm^–2^.^38^ We investigated the influence of electrode design (porosity) and cell design, including the negative-to-positive capacity balance (N/P ratio), on the rate performance and cycle life of MNO/Li half-cells and MNO/NMC622 full cells, with practical voltage limitations in use.

## 2 Experimental Section

### 2.1 Material Characterization

A scanning electron microscope (TM3030+ Benchtop SEM, Hitachi, Japan) was used to observe the size and morphology of raw anode and cathode active material powders. The microstructure of the anode and cathode electrodes and the distribution of the active material, carbon black powder, and carbon nanotubes were investigated by conducting the scanning electron microscopy (SEM) analysis of cross-sectioned anode and cathode coatings. The cross sections were performed using a Hitachi Ion Milling System (IM4000 plus) at an accelerating voltage of 5 kV for 3 h. SEM imaging was conducted on pristine (noncycled) electrode coatings at a working distance of 8 mm in an FEI Quanta scanning electron microscope under an acceleration voltage of 15 and 20 kV for the cathode and anode, respectively.

### 2.2 Electrode Mixing and Coating

The active anode material was a modified MoNb_12_O_33_ (MNO) molybdenum niobium oxide powder (developmental material, XNO, Echion Technologies Ltd., U.K.). The cathode material was a commercial grade NMC622 powder (Targray, Canada). The electrolyte used in all cells was a battery-grade solution of 1.0 M lithium hexafluorophosphate (LiPF_6_) in ethylene carbonate (EC) and diethyl carbonate (DEC) in a volume ratio of 50:50 (Sigma-Aldrich, Merck, U.K.).

Both anode and cathode electrodes were made of 90 wt % active material, 5 wt % poly(vinylidene fluoride) (PVDF) binder, and 5 wt % conductive carbon additives—of which 4.5 wt % was carbon black (TIMCAL Super C45 in anode inks and TIMCAL Super C65 in cathode inks) and 0.5 wt % were thin multiwalled carbon nanotubes (MWCNTs, NC7000, Nanocyl SA, Belgium). The carbon nanotubes were added to all anode and cathode ink formulations as it has been shown in the literature to improve the electrochemical properties and increase the electrical conductivity of battery electrodes.^39,40^

Anode and cathode active material powders were dried in a vacuum oven at 120 °C for 24 h before ink preparation. First, the carbon black powder was dispersed in PVDF and 1-methyl-2-pyrrolidinone (NMP) using an ARE 250 Thinky centrifugal mixer (2000 rpm for 3 min, repeated twice). CNTs and more NMP were then added and mixed before the progressive addition of the active material powder (mixing at 2000 rpm for 3 min between each step). Electrode inks had a powder solid loading of ∼40 wt %. Inks were coated using a draw-down coater with a micrometer adjustable doctor blade (K Paint Applicator, RK Printcoat Instruments, U.K.) at 1 cm s^–1^ onto battery-grade aluminum foil (16 μm thickness). The blade gap was set so as to obtain an active material mass loading of 5.5 ± 0.5 mg cm^–2^ for MNO anodes and 6.0 ± 0.5 mg cm^–2^ for NMC622 cathode coatings. After coating, electrodes were immediately dried at 80 °C on a hot plate and then transferred in a vacuum oven at 120 °C overnight. Dry coatings were then calendared to the desired electrode density using a hot rolling press (MSK-HRP-01, MTI Corporation, USA).

### 2.3 Cell Assembly

Calendared coatings were cut into individual electrode disks with a diameter of 14.8 and 15 mm for cathodes and anodes, respectively.

In full-coin cells, anode and cathode disks were individually matched to provide the desired negative-to-positive areal capacity ratio (N/P ratio) in each cell, assuming a rated reversible specific capacity of 170 mAh g^–1^ for NMC622 and 200 mAh g^–1^ for MNO at 20 mA g^–1^ (∼0.1 mA cm^–2^) in their respective voltage window of 2.7–4.25 and 1.1–2.4 V. In half-coin cells, lithium foil (PI-KEM) was rolled to 70 ± 10 μm thickness and cut into 15 mm discs to be used as the counter electrode.

CR2032 coin cells were prepared using the following: a single trilayer polymer Celgard H1609 separator disk with a diameter of 16 mm; 70 μL of electrolyte (∼40 μL cm^–2^); two stainless steel spacers (1 and 0.5 mm); and a 1000 psi crimping pressure. Cell components, including casing, spacers, spring, and separator, were stored in a vacuum oven at 50 °C before use.

Three-electrode PAT cells (El-Cell, France) used a double-layered PP fiber/PE membrane separator (FS-5P, 220 μm), an ultrathin Li reference ring, and were filled with 100 μL of electrolyte.

Single-layer pouch cells were manufactured using a 30 × 30 mm^2^ cathode coating and a 31 × 31 mm^2^ anode, with a Celgard H1609 separator and 380 μL of electrolyte (∼40 μL cm^–2^).

The main characteristic of our cells, including the dimensions of the positive electrode (PE) and a negative electrode (NE), is summarized in the Supporting Information (Table S1).

The entire preparation from ink mixing and electrode coating to coin and pouch cell manufacturing was performed in a dry room (dew point between −45 and −60 °C). Three-electrode cells were assembled under an argon atmosphere in a glovebox.

### 2.4 Electrochemical Analysis

After cell assembly, a resting period of at least 6 h was allowed for the electrolyte to fully soak in the porous electrodes and separator. Electrochemical testing was conducted on BCS and VMP3 battery testers (Biologic, France). Cells first underwent two CCCV formation cycles at 25 °C using a constant current density of 10 mA/g; the current was allowed to decay to 4 mA/g during the constant voltage step. Potentiostatic electrochemical impedance spectroscopy (PEIS) was performed in half-cells and full cells at ∼50% SOC after formation and at 80% SOH by using an amplitude of 5 mV in the applied frequency range of 5 × 10^5^ to 10^–2^ Hz.

Asymmetric charge and discharge rate tests were performed in half- and full cells using current densities ranging from 20 to 4000 mA g^–1^ (i.e., from ∼0.1 to ∼20 mA cm^–2^). The evolution of internal resistances and rate performance throughout the life of full cells was investigated by performing EIS and rate testing at various SOHs in coin and pouch cells.

In full cells, long-term cycling was performed at 25 °C using 200 mA g^–1^ (∼1.0 mA cm^–2^) on both charge and discharge with a short rest after each charge and discharge to observe the evolution of the IR drop. In full cells, the current density was calculated relative to the mass of cathode active material. Cycling was also performed at a higher rate on another set of cells using a discharge current density of 1000 mA g^–1^ (∼5.0 mA cm^–2^).

## 3 Results and Discussion

### 3.1 Characterization of Powders and Electrode Coatings

**Figure 1:**
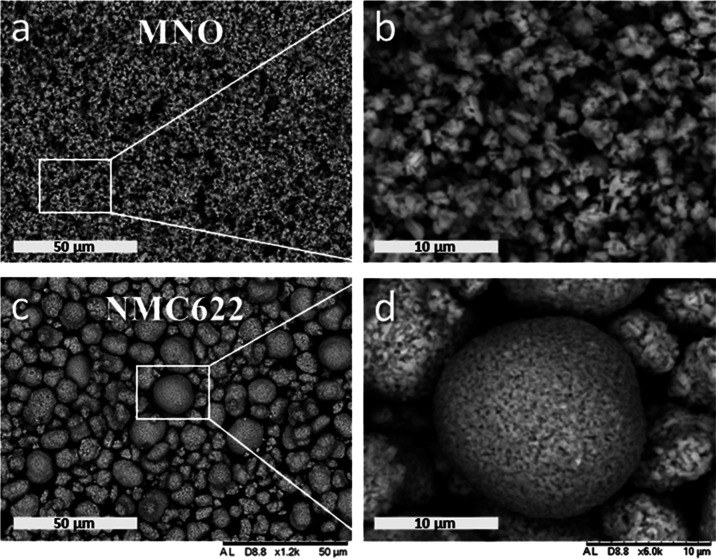
SEM images of the raw MNO anode powder composed of micron-sized particles (a, b) and the NMC622 cathode powder, which consisted of a spherical secondary particle made of nanosized primary grains (c, d).

**Figure 2:**
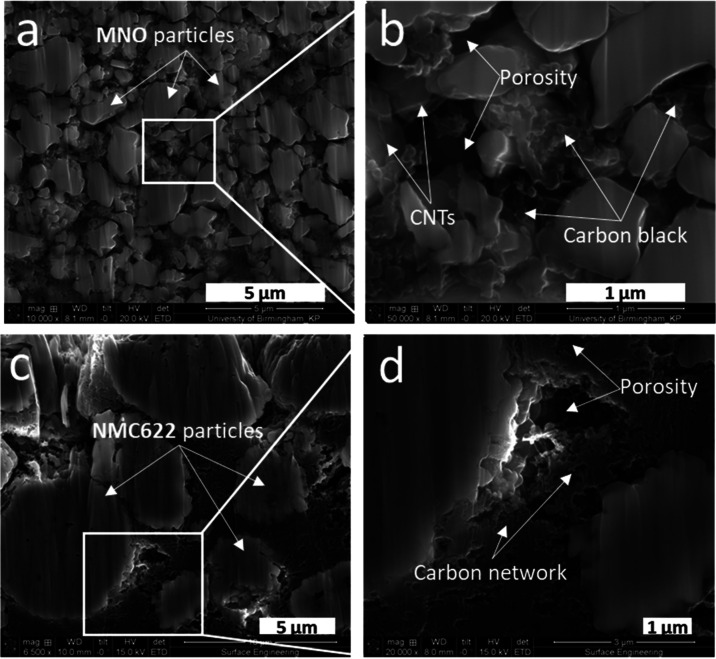
SEM images of cross-sectioned MNO (a, b) and NMC622 (c, d) electrode coatings produced in this work.

Figure 1 shows the particle morphology of the anode and cathode powders, MNO and NMC622, respectively, as observed with an SEM. These images were taken at the same magnification to enable a comparison of the size and morphology of the two active material powders. On the one hand, the MNO powder was composed of irregular-shaped micron-sized particles with an apparent diameter ranging from 1 to 4 μm (a–c). On the other hand, the NMC622 powder was composed of more spherical secondary particles with a diameter between 5 and 20 μm (d–f). Both anode and cathode electrode coatings were cross-sectioned and imaged by SEM to observe and analyze the internal pore structure and distribution of conductive carbons within the electrode matrix (Figure 2). The small micron-sized MNO powder resulted in well-distributed carbon black and CNTs throughout the electrode, while the bigger NMC622 particles led to a more continuous and denser network of carbon black.

### 3.2 MNO Electrode Porosity Optimization

**Figure 3:**
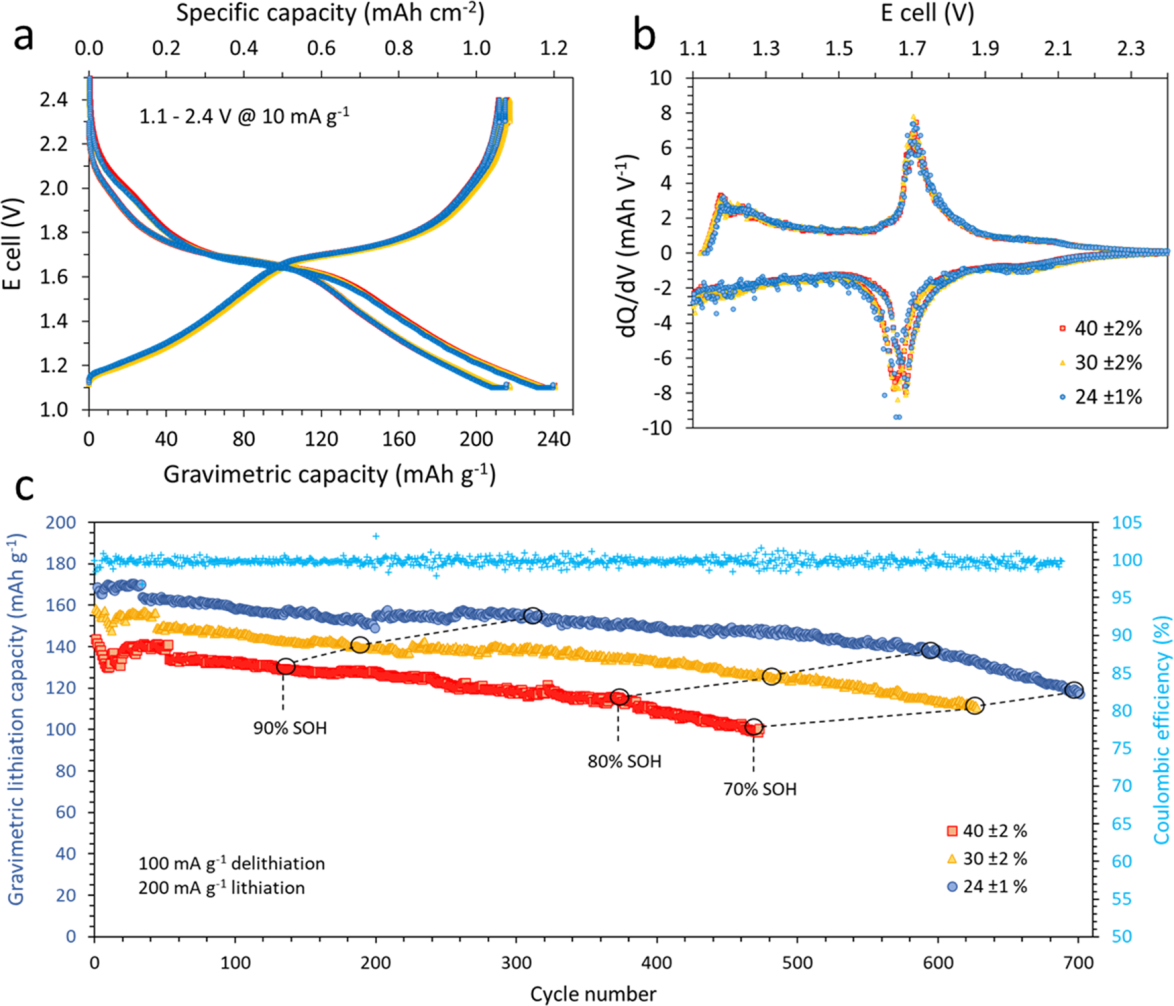
Galvanostatic cycling of MNO/Li anode half-coin cells with various electrode porosities. The first two formation cycles at 10 mA g^–1^ (a) and associated d*Q*/d*V* plots (b). Long-term asymmetric cycling with 100 mA g^–1^ charge–200 mA g^–1^ discharge (c) showing significant improvement in cycle life with increased calendaring pressures. All tests were performed at 25 °C.

The effect of electrode porosity on the electrochemical properties was studied in MNO vs Li coin cells using a series of galvanostatic cycles at various charge and discharge rates. First, cells were formed at 25 °C by performing two CCCV cycles between 1.1 and 2.4 V with a constant current density of 10 mA g^**–**1^. The capacity plot of formation is shown in Figure 3a, alongside the associated d*Q*/d*V* plot in Figure 3b. MNO electrodes delivered a high reversible capacity of up to 215 mAh g^–1^ at 10 mA g^–1^, with a Coulombic efficiency of 90.0% in the first cycle and 98.5% in the second cycle. The initial Coulombic efficiency is higher than with graphite electrodes because the potential at the anode is above the reduction potential of the electrolyte, which keeps the SEI formation to a minimum.^21,32,40^ Therefore, the primary source of the first cycle loss is likely due to irreversible intercalation and trapping of lithium ions in the crystal structure (an activation process). Side reactions with the electrolyte could still be a secondary source of first cycle loss, as is seen with the formation of SEI or SEI-like layers constituted of both organic and inorganic compounds formed at the surface of LTO.^41−43^

Figure 3c demonstrates the good long-term cyclability at 25 °C of the MNO/Li half-cells subjected to CCCV charging (delithiation) at 100 mA g^–1^ and CC discharging (lithiation) at 200 mA g^–1^ between 1.1 and 2.4 V. MNO electrodes with a 40 ± 2% porosity reached the key state-of-health levels of 90, 80, and 70% SOH after 130, 374, and 473 cycles, respectively, while reducing the porosity down to 24 ± 1% through calendering led to significant improvements, as the same SOH levels were reached after 307, 600, and 689 cycles, respectively, representing an improvement of 200 ± 25 cycles throughout the life cycle of the cell.

**Figure 4:**
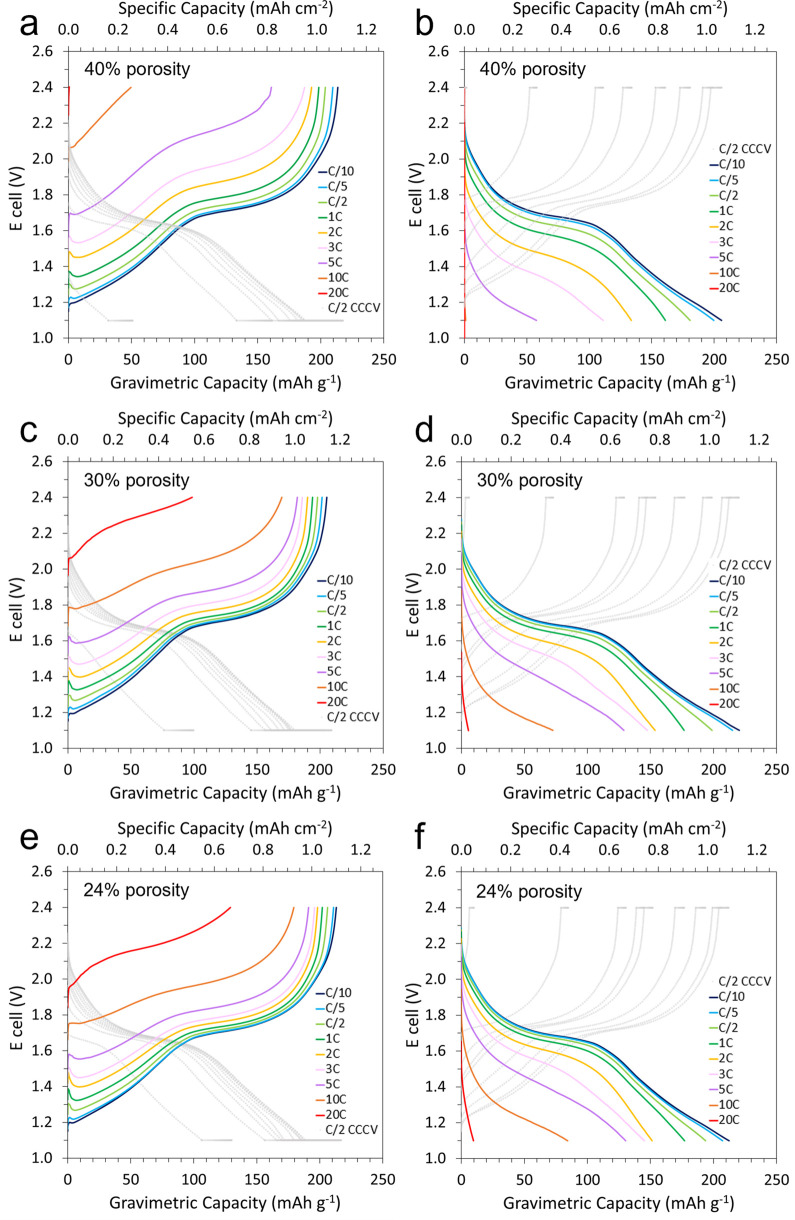
Asymmetric delithiation and lithiation rate tests of MNO half-cells with increasing C-rates from C/10 to 20C and with a constant C/2 lithiation and delithiation, respectively. Delithiation rate (a) and lithiation rate (b) with a 40% porosity MNO anode; delithiation rate (c) and lithiation rate (d) with a 30% porosity; delithiation rate (e) and lithiation rate (f) with a 24% porosity showing overall better rate performance in delithiation than in lithiation and enhanced capacity retention with decreasing anode porosity.

Asymmetric charge and discharge rate tests, respectively, were performed between 1.1 and 2.4 V by incrementally increasing the rate from C/10 (∼20 mA g^–1^) up to 20C (∼4000 mA g^–1^) while applying a constant discharge or charge rate of C/2 (∼100 mA g^–1^). Figure 4 displays the delithiation and lithiation capacities at these various C-rates for each anode porosity level. First, it appeared clearly that these anodes performed better in delithiation than in lithiation, as significantly higher capacities were obtained at all rates above C/5 in charge (a, c, e) compared with those of discharge (b, d, f), regardless of the electrode porosity level. For instance, the electrode with a 40% porosity produced 161 mAh g^–1^ on a 5C delithiation compared with only 58 mAh g^–1^ upon lithiation at the same rate. Besides, while calendaring initially had little effect on the specific capacity of MNO/Li half-cells at C/10 and C/5, it had a significant impact on capacity retention at higher rates. Indeed, as the porosity of the anode was reduced from 40 ± 2 to 30 ± 2%—i.e., an increase of the anode density from 2.6 to 3.0 g cm^–3^, which corresponded to a reduction of the thickness from 23 ± 2 to 19 ± 1 μm—the gravimetric delithiation capacity was increased from 161 to 182 mAh g^–1^ at 5C and from 1 to 98 mAh g^–1^ at 20C. Moreover, the electrode calendared to 24 ± 1% (i.e., density of 3.3 g cm^–3^ and thickness of 17 ± 1 μm) displayed further improvements in capacity retention with 191 mAh g^–1^ at 5C and 129 mAh g^–1^ at 20C. Therefore, the 24% porosity electrode displayed exceptional delithiation rate performance with a 93% capacity retention at 5C (relative to the capacity at C/2) and as much as 87 and 63% capacity retention at 10C and 20C, respectively. Lithiation rate performance was still excellent although lower than in delithiation, with 67 and 44% capacity retention at 5C and 10C, respectively. However, the lithiation capacity was only 10 mAh g^–1^ at 20C (∼20 mA cm^–2^); this was likely due to current rate limitations in half-cells caused by the use of lithium metal with a liquid organic electrolyte, as described in the literature.^44,45^ Therefore, resistances and rate performance were further investigated in full-cell setups.

An explanation for the improved performance of electrodes with low porosity could be that the increased calendaring pressure results in decreased porosity and better particle packing, which improves connectivity between the carbon additive and active material phases, removing local bottlenecks in electrical conductivity. Another explanation could be that the decreased thickness reduces the average ionic and electronic path lengths. Both effects seem to weigh up against the effect that the lower porosity would increase the tortuosity of the ionic path in the electrolyte and that it increases the chance of local bottlenecks in the electrolyte.

A similar investigation of the electrochemical behavior, rate performance, and cycle life of NMC622/Li half-cells as a function of electrode porosity was also conducted to provide a benchmark of the cathode properties for subsequent full cells. The results are provided in the Supporting Information (Figure S1) alongside PEIS results for both MNO/Li and NMC622/Li half-cells (Figure S2).

### 3.3 Three-Electrode Full Cells: Insight into Individual Electrode Potentials

**Figure 5:**
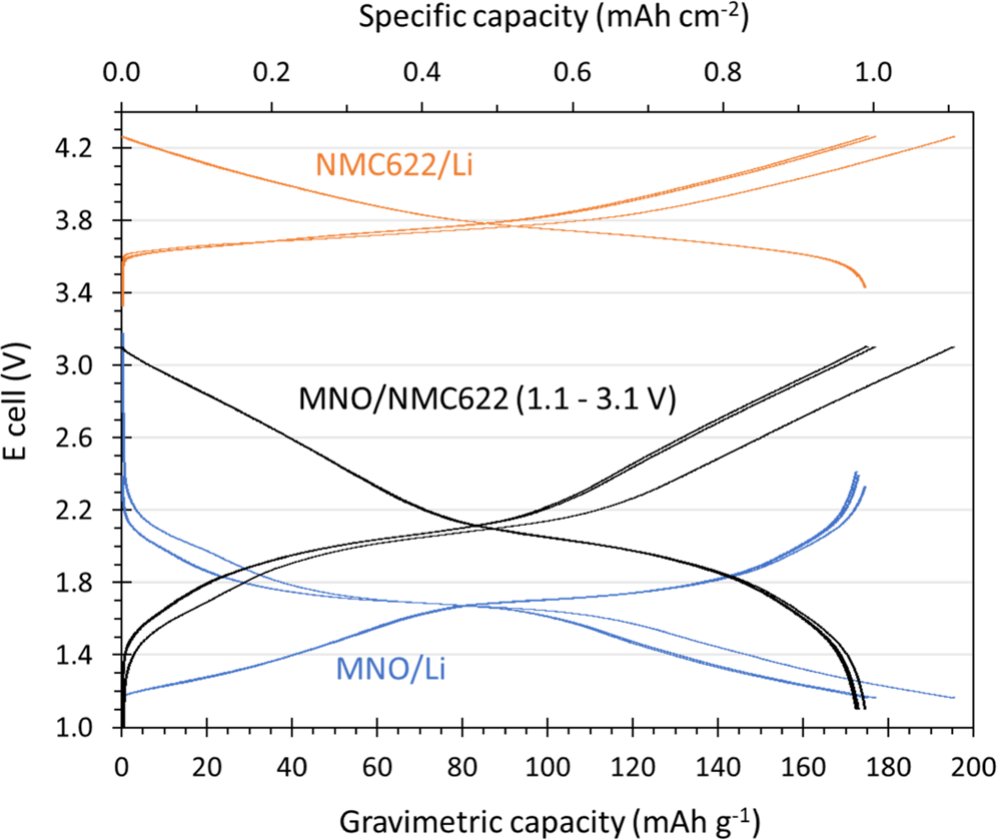
Formation curves of a three-electrode PAT cell with NMC622, ECA211, and Li metal as the cathode, anode, and reference electrode, respectively.

A three-electrode full cell was constructed in an argon glovebox to obtain a better understanding of the individual potentials reached at the anode and cathode, respectively, when cycling an MNO/NMC622 full cell (N/P ratio of 1.1) within the chosen voltage window of 1.1–3.1 V. The three formation cycles of the three-electrode full cell are displayed in Figure 5. On the one hand, as the full cell was charged to 3.1 V, the NMC622 cathode reached 4.27 V, while the potential at the MNO anode fell to 1.16 V. This was ideal as (i) the maximum potential at the cathode should ideally be below 4.3–4.4 V to avoid excessive O_2_ release upon the oxidation of Ni and prevent microcrack generation and particle surface degradation due to anisotropic lattice changes;^46^ (ii) the potential at the anode should remain above 1.0 V as it is thought to limit the growth of the SEI layer on niobate anode. As the full cell was discharged to 1.1 V, the potential at the cathode and anode reached 3.4 and 2.4 V, respectively, where both appeared to be ideal from the shape of the formation curves. It can be observed that there was a slight voltage creep at both the anode and the cathode from one discharge to the next, with the potential at the anode increasing from 2.33 to 2.39 V and then 2.41 V during the first, second, and third cycles, respectively, while the potential at the cathode reached 3.43, 3.49, and 3.51 V. This “voltage walking” could be explained by lithium inventory loss, where Li^+^ extracted from the cathode during charge remains permanently within the anode, thus incurring a loss of cyclable Li^+^, coupled with the loss of active anode and cathode sites due to structural disordering.

### 3.4 Electrode Mass Balancing

Full-coin cells were constructed by individually matching MNO anodes and NMC622 cathodes to obtain the desired N/P ratio for each cell. The N/P capacity ratio was varied between 0.9 and 1.2 for two C/20 formation cycles followed by 1C/1C cycling tests at 25 °C. Further cycling was then performed using selected N/P ratios at faster charge/discharge rates, including 1C/5C and 2C/2C.

Figure S3 in the Supporting Information shows the galvanostatic charge–discharge formation curves and their associated d*Q*/d*V* graph of multiple MNO/NMC622 full-coin cells with N/P ratios of 0.9, 1.0, 1.1, and 1.2 cycled at 10 mA g^–1^ between 1.1 and 3.1 V. The N/P ratio had no impact on the overall shape of the formation curves, including the first cycle loss, polarization, and location and amplitude of peaks on the d*Q*/d*V* plots. However, it appears clearly that a higher specific capacity was obtained as the N/P ratio was increased. Indeed, a reversible capacity of 160, 165, 170, and 180 mAh g^–1^ was obtained for N/P ratios of 0.9, 1.0, 1.1, and 1.2, respectively. The FCL was constant across all N/P ratios at ∼12%.

The d*Q*/d*V* plots in Figure S3 show that upon the first charge, all full cells displayed a small peak at 1.65 V followed by a much larger peak with an amplitude of 4 mAh V^–1^ at ∼2.1 V. Specifically, the 1.65 V peak loses intensity after the first cycle, which indicates that the intercalation process at this voltage is irreversible. Also, the amplitude of the 2.1 V peak reduces to 3 mAh V^–1^, which indicates that intercalation is not fully symmetrical and that irreversible loss can also occur at other stages in the intercalation process.

**Figure 6:**
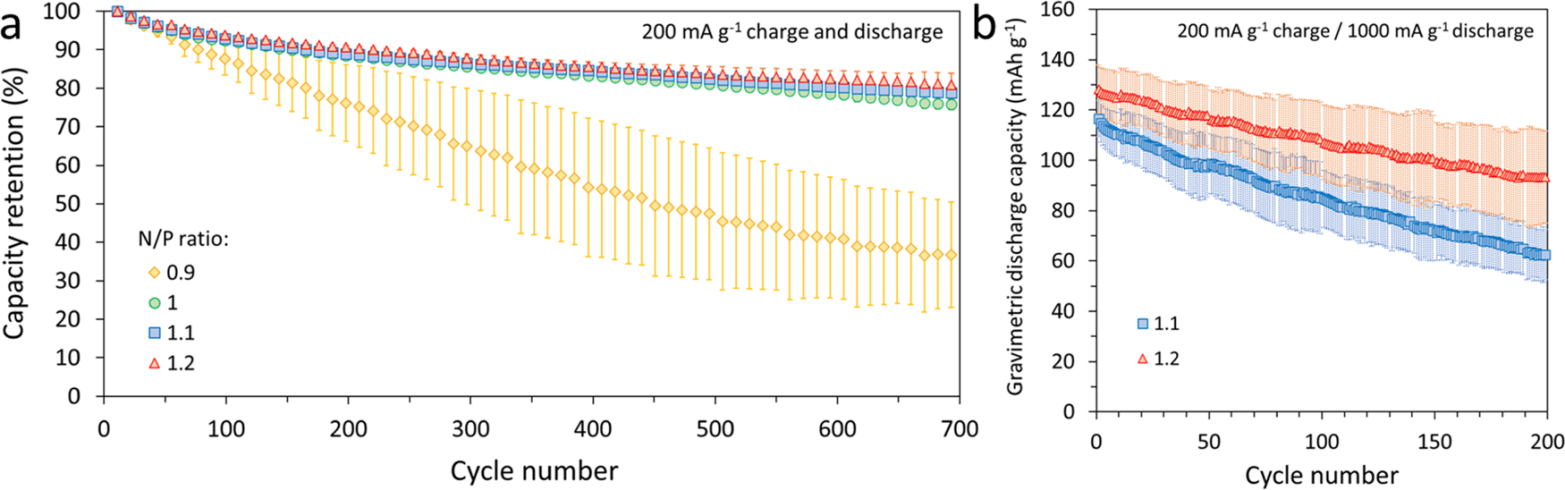
Evolution of the discharge capacity of MNO/NMC622 full-coin cells at 25 °C with various N/P ratios cycled using 200 mA g^–1^ charge/discharge (a) and when cycled using 200 mA g^–1^ charge and 1000 mA g^–1^ discharge (b).

**Figure 7:**
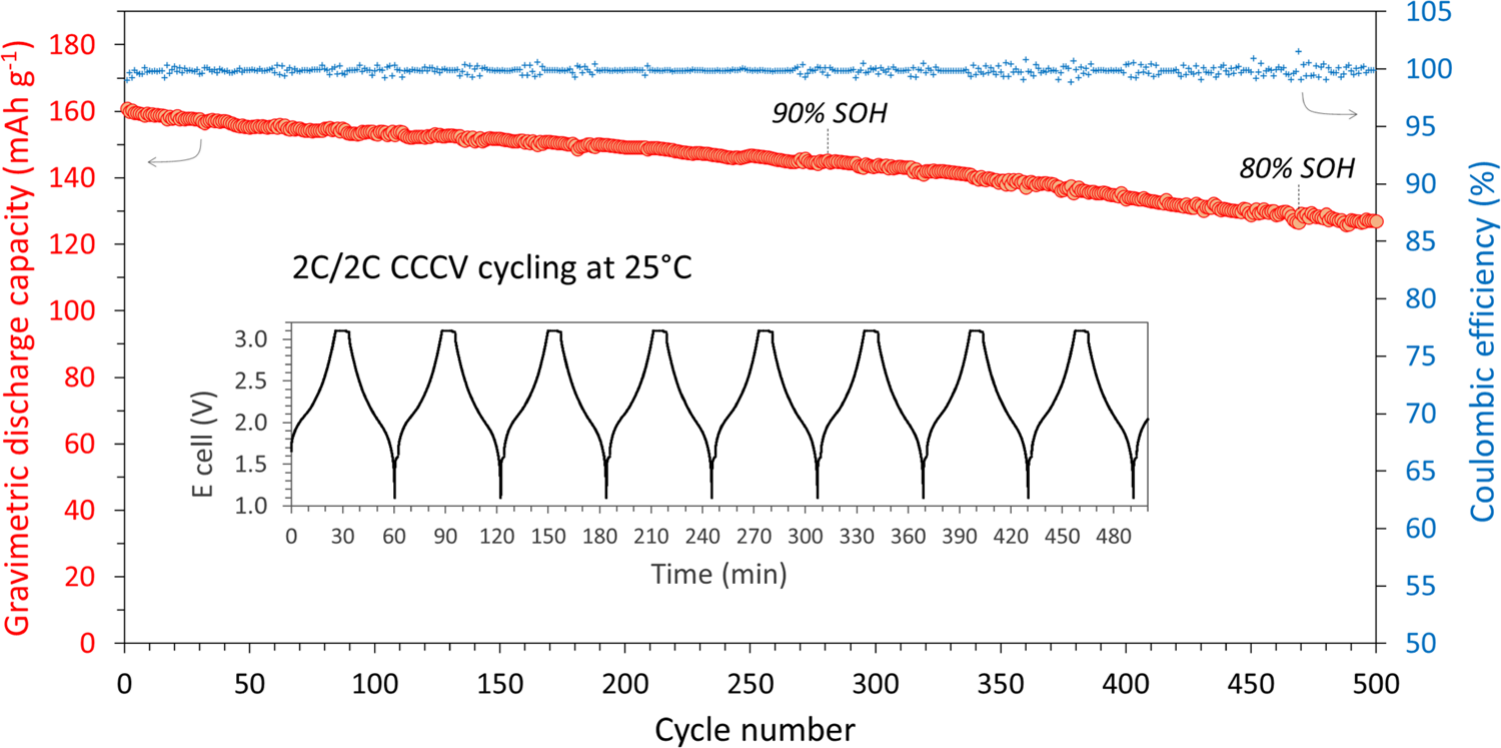
2C/2C cycling (CCCV charge, CC discharge) of MNO/NMC622 full-coin cells at 25 °C.

After formation, MNO/NMC622 full-coin cells were cycled using a 200 mA g^–1^ current density on both charge and discharge at 25 °C. Figure 6a shows the capacity retention of full cells over 700 cycles as the N/P ratio was increased from 0.9 to 1.2. Figure 6b shows the evolution of the specific capacity of full cells with N/P ratios of 1.1 and 1.2 cycled at a higher rate than previously using a 200 mA g^–1^ charge and 1000 mA g^–1^ discharge. Full cells experienced materially faster capacity fade at this higher rate of discharge, reaching 54% SOH on average after only 200 cycles for cells with an N/P ratio of 1.1, while cells with an N/P ratio of 1.2 degraded slower, reaching 72% SOH on average after 200 cycles. Nevertheless, Figure 7 shows that optimized coin cells with an N/P ratio of 1.2 cycled at a 2C rate of charge and discharge reached 470 cycles to 80% SOH.

### 3.5 Demonstration of MNO/NMC622 Full Cells

**Figure 8:**
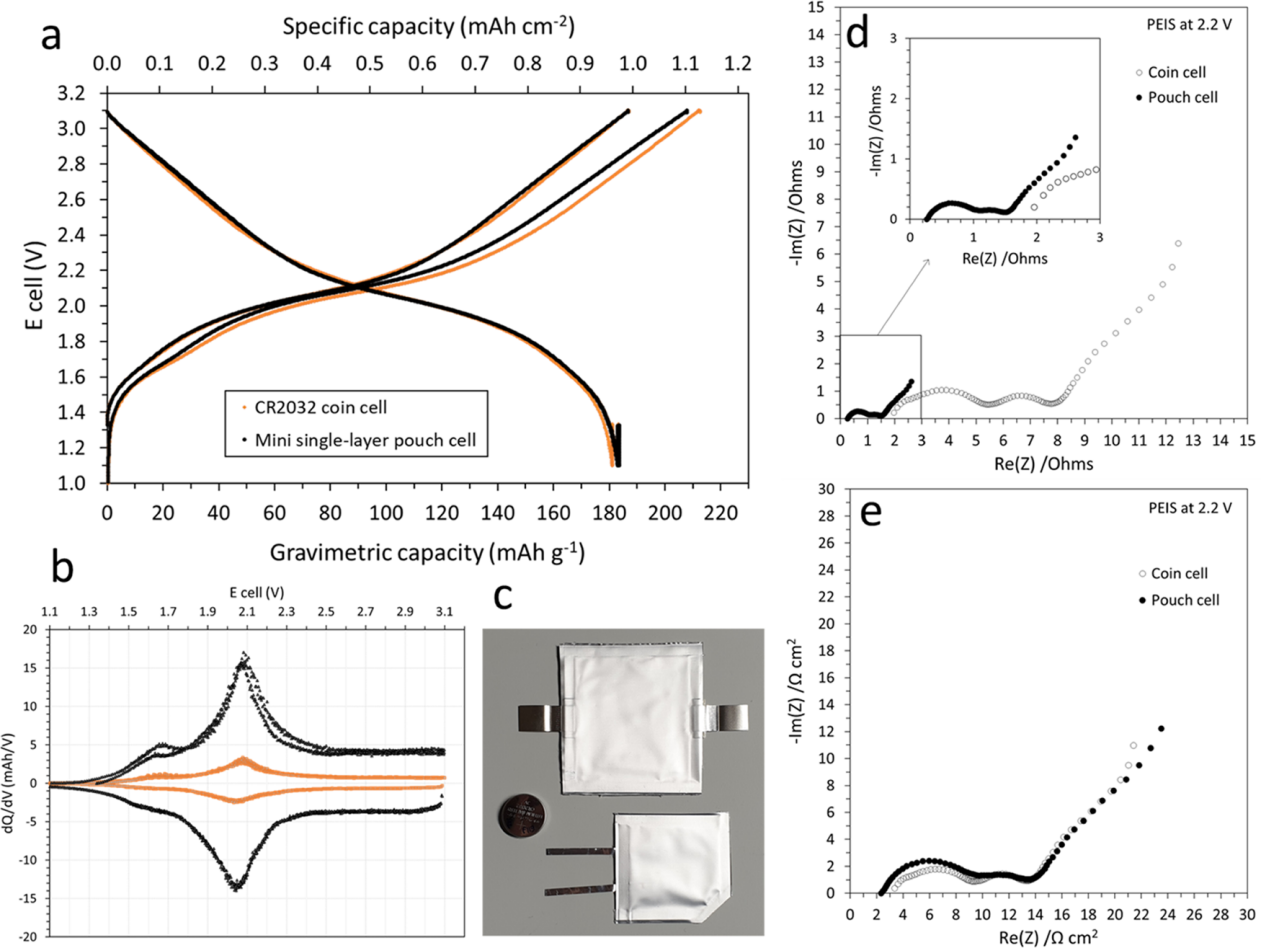
Capacity curve of formation (a) and associated d*Q*/d*V* plots (b) for MNO/NMC622 in CR2032 coin cell and small pouch cell formats (c). Nyquist plots from PEIS performed on both cell formats after formation expressed in Ω (d) and in Ω cm^2^ (e). PEIS was performed using a 5 mV amplitude in the frequency range of 500 kHz to 10 mHz.

A full-coin cell and a small single-layer pouch cell with optimized electrode porosities (24 ± 1%) and N/P ratio (1.2) were constructed and tested to evaluate the influence of the cell format on the rate performance and cycle life of MNO/NMC622 full cells (Figure 8). A reversible gravimetric capacity of ∼180 mAh g^–1^ was obtained with both cell formats; the FCL in the small pouch cell was ∼2% lower than in the coin cell. Furthermore, postformation PEIS performed at 2.2 V in both cell formats revealed the remarkably lower internal resistance in the pouch cell, although the Nyquist plots were almost identical when expressing the resistances in Ω cm^2^ rather than in Ω.

**Figure 9:**
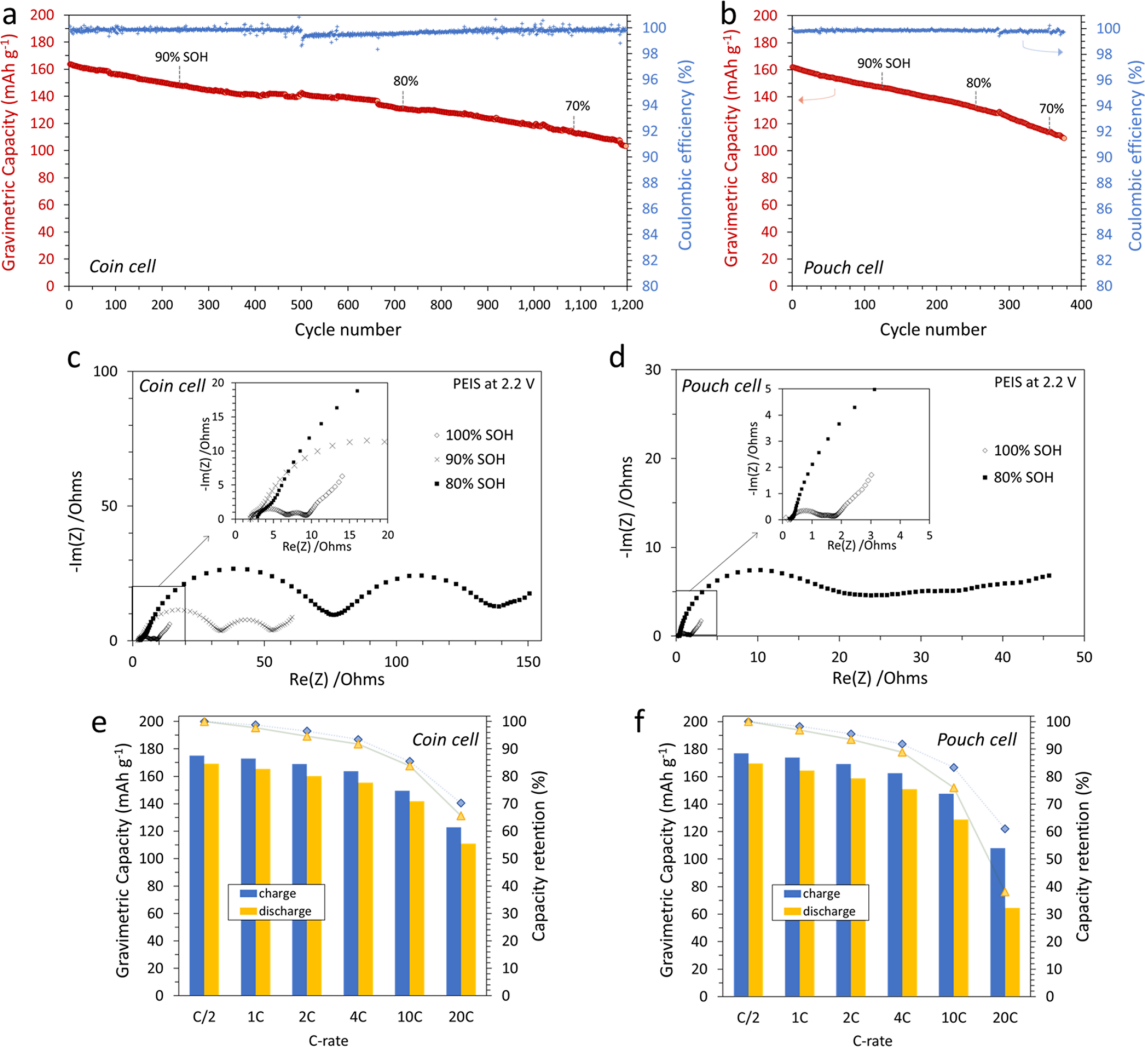
1C/1C cycling of MNO/NMC622 in CR2032 optimized full-coin cell (a) and in small prototype single-layer pouch cell (b). PEIS performed at 2.2 V between 100 and 80% SOH in coin cell (c) and pouch cell (d). Comparison of results from asymmetric charge and discharge rate testing of MNO/NMC622 in coin cell (e) and in small single-layer pouch cell (f). Testing was performed at 25 °C.

Full-coin cells displayed excellent cyclability at 200 mA g^–1^ charge–discharge, reaching 80% SOH after 720 cycles and 70% SOH after 1080 cycles (Figure 9a). However, faster capacity fade was observed in the small pouch cell configuration compared to coin cells due to unoptimized pouch cell design (Figure 9b). This faster capacity fade was also highlighted by the distortion of the Nyquist plot obtained from PEIS at 80% SOH in pouch cell compared to the optimized coin cell (Figure 9c,d). Moreover, Figure 9e,f shows that MNO/NMC622 full cells displayed outstanding charge and discharge rate performances regardless of the cell type, although the lack of optimization of the small pouch cells led to lower capacity retention at high rates. Indeed, the capacity upon charging at 100 mA g^–1^ was 179 mAh g^–1^; the capacity at 800 mA g^–1^ was 164 mAh g^–1^ and it was as high as 150 mAh g^–1^ at 2000 mA g^–1^ and 123 mAh g^–1^ at 4000 mA g^–1^. Therefore, MNO/NMC622 cells could be charged from 0 to 92% capacity in as little as 12 min and to 70% capacity in ∼120 s at 4 A g^–1^ (∼20 mA cm^–2^). The full-cell rate capability on charge is significantly better than in lithiation of the MNO/Li half-cells displayed in Figures 3 and 4, which confirms that the main limiting factor in half-cells is the Li-metal counter electrode, while the MNO and NMC622 have excellent high-rate capability in both charge and discharge.

### 3.6 Discussion

In this work, we characterized and tested lithium-ion half- and full cells based on a novel niobium oxide anode material: a micron-sized, modified MoNb_12_O_33_. Electrochemical tests were carried out in half- and full-coin cells, MNO/Li and MNO/NMC622, respectively, as well as in three-electrode PAT cell and small single-layer pouch cell formats.

First, results obtained from NMC622/Li half-coin cell testing were in line with the literature and showed the excellent performance of this material in terms of both high-rate performance and cyclability. The extent of electrode calendaring had no significant influence on neither the rate performance nor the cycle life of NMC622 cathodes, although the initial Coulombic efficiency and absolute gravimetric capacity could be increased significantly by reducing the electrode porosity. On the other hand, the rate performance of MNO/Li half-coin cells depended strongly on electrode porosity, with significantly better capacity retention at a high rate for electrodes that were calendared down to 24 ± 1% porosity (density of 3.3 g cm^–1^, thickness of 17 ± 1 μm) compared to electrodes with 30 ± 2% (3.0 g cm^–1^, 19 ± 1 μm) and 40 ± 2% porosity (2.6 g cm^–1^, 23 ± 2 μm). Furthermore, anode porosity also affected the cyclability of MNO electrodes at 25 °C, and the most porous MNO electrode reached 80% SOH after only 374 cycles at 200 mA g^–1^, while the denser electrode reached 600 cycles to 80% SOH. The difference between NMC622 and MNO in sensitivity to porosity could be explained by their difference in electrode microstructure, as observed in the SEM images in Figure 2. The less hierarchically organized MNO electrode has more risk of local bottlenecks in the electrical path and therefore benefits more from compaction.

MNO/Li half-coin cells using a 24% porous electrode exhibited outstanding rate performance in delithiation with as much as 87% capacity retention at 10C and 63% at 20C (i.e., at current densities of ∼10 and 20 mA cm^–2^, respectively). Lithiation rate performance was also excellent although lower than in delithiation. However, this was resolved when the Li disk was replaced by an NMC622 cathode to form full cells. Indeed, in both coin and single-layer pouch MNO/NMC622 full cells, asymmetric rate testing showed superior capacity retention at high rates of both charge and discharge, with 84% retention of capacity at 2 A g^–1^ (wrt NMC) in full-coin cells. MNO/NMC622 cells could be charged from 0 to 92% capacity in as little as 12 min and to 70% capacity in ∼120 s at 4 A g^–1^ (∼20 mA cm^–2^). However, while scaling up from a CR2032 coin cell to a 30 × 30 mm^2^ single-layer pouch cell resulted in materially lower impedance and internal resistances, it also led to accelerated capacity fade as 350 cycles corresponded to 90% SOH in coin cell compared to 70% SOH in pouch cell. This is likely due to the electrolyte quantity and degradation reactions, with less excess of electrolyte in the pouch cell due to excess packaging volume and surface area of separator in pouch cells compared with tight coin cells (folded separator in pouch cell result in more than 3 times more separator-to-electrode surface ratio than in coin cell), as well as a much more accurate electrolyte deposition method in coin cells than in pouch cells. This, however, needs further investigation. Full-coin cells with various N/P ratios were also cycled at 25 °C at ∼1C/1C and 1C/5C charge and discharge rates (respectively, 200/200 and 200/1000 mA g^–1^ wrt NMC), showing excellent cyclability at 200 mA g^–1^ but accelerated capacity fade with the five times faster discharge rate, with a significant improvement in cycle life with an N/P ratio of 1.2 compared with that of 0.9–1.1.

Future works need to focus on the effect of the electrolyte upon cycling, particularly the electrolyte concentration, degradation reactions, and quantity. The increased electrolyte ratio in the coin cell likely improved cycled life when compared to the pouch cell, and optimization of the pouch cell design needs further investigation. In addition, the improved understanding of the electrochemical diffusion kinetics and thermodynamics of this MNO material and electrodes is required for further optimization. The impact of the cycling temperature, as well as the influence of higher mass loadings (areal capacity of 2–4 mAh cm^–2^), on the rate performance and calendar life of MNO should also be characterized. It would be ideal to further investigate full cells in single- and multilayer pouch cell configurations to better understand the various implications of these scaled-up cell geometries on the electrochemical behavior of niobate anodes. Finally, it would be extremely interesting to investigate how switching from the PVDF/NMP system to a more sustainable and cost-effective aqueous binder system would impact the electrochemical performance of MNO electrodes.

## 4 Conclusions

To conclude, a high-rate molybdenum niobium oxide vs NMC622 in full-cell configuration is demonstrated for the first time at coin cell and single-layer pouch scale. Through careful electrode and cell design optimization and reduction of internal resistances of cell formats, improved cycle life and rate capabilities are observed. Porosity, in particular, had a significant impact on the rate performance of MNO half-cells, and performance was much improved upon electrode calendaring, as the micron size of this MNO powder resulted in easier calendaring compared to competing anodes such as nanosized LTO. Regarding the full-cell design and N/P ratio, a higher specific capacity as well as improved rate and cycling performance were obtained in this work with an N/P ratio of 1.2 for MNO/NMC622 using a standard 1 M LiPF_6_ in 50:50 EC/DEC electrolyte. Charge rates of 2 min to 70% capacity were observed and over 1000 cycles to 70% of initial capacity at 1C rate of charge and discharge.

## Supporting Information Available

Half- and full-cell building details, including information on positive electrodes (PEs), negative electrodes (NEs), separators, and electrolyte volume (Table S1); galvanostatic cycling of NMC622/Li cathode half-cells (Figure S1); PEIS plots of the modified MoNb12O33/Li anode and the NMC622/Li cathode half-cells (Figure S2); and formation profile and d*Q*/d*V* plots of MNO/NMC622 full-coin cells (Figure S3) (PDF)

## References

[ref1] Deng D. Li-Ion Batteries: Basics, Progress, and Challenges. Energy Sci. Eng. 2015, 3, 385–418. 10.1002/ese3.95.

[ref2] Massé R. C.; Liu C.; Li Y.; Mai L.; Cao G. Energy Storage through Intercalation Reactions: Electrodes for Rechargeable Batteries. Natl. Sci. Rev. 2017, 4, 26–53. 10.1093/nsr/nww093.

[ref3] Nitta N.; Wu F.; Lee J. T.; Yushin G. Li-Ion Battery Materials: Present and Future. Mater. Today 2015, 18, 252–264. 10.1016/j.mattod.2014.10.040.

[ref4] Zhang Y. Di.; Li Y.; Xia X. H.; Wang X. L.; Gu C. D.; Tu J. P. High-Energy Cathode Materials for Li-Ion Batteries: A Review of Recent Developments. Sci. China Technol. Sci. 2015, 58, 1809–1828. 10.1007/s11431-015-5933-x.

[ref5] Zhang Y.; Wang C.-Y. Cycle-Life Characterization of Automotive Lithium-Ion Batteries with LiNiO[Sub 2] Cathode. J. Electrochem. Soc. 2009, 156, A527 10.1149/1.3126385.

[ref6] Pinson M. B.; Bazant M. Z. Theory of SEI Formation in Rechargeable Batteries: Capacity Fade, Accelerated Aging and Lifetime Prediction. J. Electrochem. Soc. 2013, 160, A243–A250. 10.1149/2.044302jes.

[ref7] An S. J.; Li J.; Daniel C.; Mohanty D.; Nagpure S.; Wood D. L. The State of Understanding of the Lithium-Ion-Battery Graphite Solid Electrolyte Interphase (SEI) and Its Relationship to Formation Cycling. Carbon 2016, 105, 52–76. 10.1016/j.carbon.2016.04.008.

[ref8] Crowther O.; West A. C. Effect of Electrolyte Composition on Lithium Dendrite Growth. J. Electrochem. Soc. 2008, 155, A806 10.1149/1.2969424.

[ref9] Maraschky A.; Akolkar R. Temperature Dependence of Dendritic Lithium Electrodeposition: A Mechanistic Study of the Role of Transport Limitations within the SEI. J. Electrochem. Soc. 2020, 167, 062503 10.1149/1945-7111/ab7ce2.

[ref10] Li S.; Wang K.; Zhang G.; Li S.; Xu Y.; Zhang X.; Zhang X.; Zheng S.; Sun X.; Ma Y. Fast Charging Anode Materials for Lithium-Ion Batteries: Current Status and Perspectives. Adv. Funct. Mater. 2022, 32, 2200796 10.1002/adfm.202200796.

[ref11] Gao X.; Zhou Y. N.; Han D.; Zhou J.; Zhou D.; Tang W.; Goodenough J. B. Thermodynamic Understanding of Li-Dendrite Formation. Joule 2020, 4, 1864–1879. 10.1016/j.joule.2020.06.016.

[ref12] Jiao M.; Wang Y.; Ye C.; Wang C.; Zhang W.; Liang C. High-Capacity SiOx (0≤x≤2) as Promising Anode Materials for next-Generation Lithium-Ion Batteries. J. Alloys Compd. 2020, 842, 155774 10.1016/j.jallcom.2020.155774.

[ref13] Guo Z.; Yao H. Thickness Gradient Promotes the Performance of Si-Based Anode Material for Lithium-Ion Battery. Mater. Des. 2020, 195, 108993 10.1016/j.matdes.2020.108993.

[ref14] Xiong Y.; Xing H.; Fan Y.; Wei Y.; Shang J.; Chen Y.; Yan J. SiOx-Based Graphite Composite Anode and Efficient Binders: Practical Applications in Lithium-Ion Batteries. RSC Adv. 2021, 11, 7801–7807. 10.1039/d0ra10283k. 35423327 PMC8695106

[ref15] He S.; Huang S.; Wang S.; Mizota I.; Liu X.; Hou X. Considering Critical Factors of Silicon/Graphite Anode Materials for Practical High-Energy Lithium-Ion Battery Applications. Energy Fuels 2021, 35, 944–964. 10.1021/acs.energyfuels.0c02948.

[ref16] Moyassari E.; Streck L.; Paul N.; Trunk M.; Neagu R.; Chang C.-C.; Hou S.-C.; Märkisch B.; Gilles R.; Jossen A. Impact of Silicon Content within Silicon-Graphite Anodes on Performance and Li Concentration Profiles of Li-Ion Cells Using Neutron Depth Profiling. J. Electrochem. Soc. 2021, 168, 020519 10.1149/1945-7111/abe1db.

[ref17] Ghosh A.; Ghamouss F. Role of Electrolytes in the Stability and Safety of Lithium Titanate-Based Batteries. Front. Mater. 2020, 7, 186 10.3389/fmats.2020.00186.

[ref18] Horiba T. Lithium-Ion Battery Systems. Proc. IEEE 2014, 102, 939–950. 10.1109/JPROC.2014.2319832.

[ref19] Xu J.; Ding W.; Yin G.; Tian Z.; Zhang S.; Hong Z.; Huang F. Capacitive Lithium Storage of Lithiated Mesoporous Titania. Mater. Today Energy 2018, 9, 240–246. 10.1016/j.mtener.2018.05.016.

[ref20] Zhang H.; Deng Q.; Mou C.; Huang Z.; Wang Y.; Zhou A.; Li J. Surface Structure and High-Rate Performance of Spinel Li4Ti 5O12 Coated with N-Doped Carbon as Anode Material for Lithium-Ion Batteries. J. Power Sources 2013, 239, 538–545. 10.1016/j.jpowsour.2013.03.013.

[ref21] Yang Y.; Zhao J. Wadsley–Roth Crystallographic Shear Structure Niobium-Based Oxides: Promising Anode Materials for High-Safety Lithium-Ion Batteries. Adv. Sci. 2021, 8, 2004855 10.1002/advs.202004855. PMC8224428 34165894

[ref22] Cava R. J.; Murphy D. W.; Zahurak S. M. Lithium Insertion in Wadsley-Roth Phases Based on Niobium Oxide. J. Electrochem. Soc. 1983, 130, 2345–2351. 10.1149/1.2119583.

[ref23] Han J. T.; Huang Y. H.; Goodenough J. B. New Anode Framework for Rechargeable Lithium Batteries. Chem. Mater. 2011, 23, 2027–2029. 10.1021/cm200441h.

[ref24] Cheng Q.; Liang J.; Zhu Y.; Si L.; Guo C.; Qian Y. Bulk Ti2Nb10O29 as Long-Life and High-Power Li-Ion Battery Anodes. J. Mater. Chem. A 2014, 2, 17258–17262. 10.1039/c4ta04184d.

[ref25] Fu Q.; Li R.; Zhu X.; Liang G.; Luo L.; Chen Y.; Lin C.; Zhao X. S. Design, Synthesis and Lithium-Ion Storage Capability of Al0.5Nb24.5O62. J. Mater. Chem. A 2019, 7, 19862–19871. 10.1039/c9ta04644e.

[ref26] Yu H.; Zhang J.; Zheng R.; Liu T.; Peng N.; Yuan Y.; Liu Y.; Shu J.; Wang Z. B. The Journey of Lithium Ions in the Lattice of PNb9O25. Mater. Chem. Front. 2020, 4, 631–637. 10.1039/c9qm00694j.

[ref27] Saritha D.; Pralong V.; Varadaraju U. V.; Raveau B. Electrochemical Li Insertion Studies on WNb12O33-A Shear ReO3 Type Structure. J. Solid State Chem. 2010, 183, 988–993. 10.1016/j.jssc.2010.03.003.

[ref28] Zhu X.; Xu J.; Luo Y.; Fu Q.; Liang G.; Luo L.; Chen Y.; Lin C.; Zhao X. S. MoNb12O33 as a New Anode Material for High-Capacity, Safe, Rapid and Durable Li+ Storage: Structural Characteristics, Electrochemical Properties and Working Mechanisms. J. Mater. Chem. A 2019, 7, 6522–6532. 10.1039/c9ta00309f.

[ref29] Ran F.; Cheng X.; Yu H.; Zheng R.; Liu T.; Li X.; Ren N.; Shui M.; Shu J. Nano-Structured GeNb18O47 as Novel Anode Host with Superior Lithium Storage Performance. Electrochim. Acta 2018, 282, 634–641. 10.1016/j.electacta.2018.06.109.

[ref30] Yang C.; Yu S.; Lin C.; Lv F.; Wu S.; Yang Y.; Wang W.; Zhu Z. Z.; Li J.; Wang N.; Guo S. Cr0.5Nb24.5O62 Nanowires with High Electronic Conductivity for High-Rate and Long-Life Lithium-Ion Storage. ACS Nano 2017, 11, 4217–4224. 10.1021/acsnano.7b01163. 28358508

[ref31] Lou X.; Li R.; Zhu X.; Luo L.; Chen Y.; Lin C.; Li H.; Zhao X. S. New Anode Material for Lithium-Ion Batteries: Aluminum Niobate (AlNb 11 O 29). ACS Appl. Mater. Interfaces 2019, 6089–6096. 10.1021/acsami.8b20246. 30714359

[ref32] Spada D.; Albini B.; Galinetto P.; Versaci D.; Francia C.; Bodoardo S.; Bais G.; Bini M. FeNb11O29, Anode Material for High-Power Lithium-Ion Batteries: Pseudocapacitance and Symmetrisation Unravelled with Advanced Electrochemical and in Situ/Operando Techniques. Electrochim. Acta 2021, 393, 139077 10.1016/j.electacta.2021.139077.

[ref33] Koçer C. P.; Griffith K. J.; Grey C. P.; Morris A. J. Cation Disorder and Lithium Insertion Mechanism of Wadsley-Roth Crystallographic Shear Phases from First Principles. J. Am. Chem. Soc. 2019, 141, 15121–15134. 10.1021/jacs.9b06316. 31448601

[ref34] Groombridge A. S.; Verpilliere J. D. L.; Santhanam S.; Zhang W.; Houck M. E. Li/Na-Ion Battery Anode Materials. GB2588254A, 2021.

[ref35] Johansson P.; Alvi S.; Ghorbanzade P.; Karlsmo M.; Loaiza L.; Thangavel V.; Westman K.; Årén F. Ten Ways to Fool the Masses When Presenting Battery Research. Batteries Supercaps 2021, 4, 1785–1788. 10.1002/batt.202100154.

[ref36] Yu H.; Zhang J.; Xia M.; Deng C.; Zhang X.; Zheng R.; Chen S.; Shu J.; Wang Z. B. PNb9O25 Nanofiber as a High-Voltage Anode Material for Advanced Lithium Ions Batteries. J. Mater. 2020, 6, 781–787. 10.1016/j.jmat.2020.07.003.

[ref37] Firdous N.; Arshad N.; Simonsen S. B.; Kadirvelayutham P.; Norby P. Advanced Electrochemical Investigations of Niobium Modified Li2ZnTi3O8 Lithium Ion Battery Anode Materials. J. Power Sources 2020, 462, 228186 10.1016/j.jpowsour.2020.228186.

[ref38] Lain M. J.; Brandon J.; Kendrick E. Design Strategies for High Power vs. High Energy Lithium Ion Cells. Batteries 2019, 5, 64 10.3390/batteries5040064.

[ref39] Fan X.-m.; Zhang X.; Hu G.; Zhang B.; He Z.; Li Y.; Zheng J. Single-Walled Carbon Nanotube as Conductive Additive for SiO/C Composite Electrodes in Pouch-Type Lithium-Ion Batteries. Ionics 2020, 26, 1721–1728. 10.1007/s11581-019-03391-w.

[ref40] Aghamohammadi H.; Hassanzadeh N.; Eslami-Farsani R. A Review Study on Titanium Niobium Oxide-Based Composite Anodes for Li-Ion Batteries: Synthesis, Structure, and Performance. Ceram. Int. 2021, 47, 26598–26619. 10.1016/j.ceramint.2021.06.127.

[ref41] Dedryvère R.; Foix D.; Franger S.; Patoux S.; Daniel L.; Gonbeau D. Electrode/Electrolyte Interface Reactivity in High-Voltage Spinel LiMn1.6Ni0.4O4/Li4Ti5O12 Lithium-Ion Battery. J. Phys. Chem. C 2010, 114, 10999–11008. 10.1021/jp1026509.

[ref42] He Y. B.; Liu M.; Huang Z. D.; Zhang B.; Yu Y.; Li B.; Kang F.; Kim J. K. Effect of Solid Electrolyte Interface (SEI) Film on Cyclic Performance of Li4Ti5O12 Anodes for Li Ion Batteries. J. Power Sources 2013, 239, 269–276. 10.1016/j.jpowsour.2013.03.141.

[ref43] Gauthier N.; Courrèges C.; Demeaux J.; Tessier C.; Martinez H. Probing the In-Depth Distribution of Organic/Inorganic Molecular Species within the SEI of LTO/NMC and LTO/LMO Batteries: A Complementary ToF-SIMS and XPS Study. Appl. Surf. Sci. 2020, 501, 144266 10.1016/j.apsusc.2019.144266.

[ref44] Jiang Z.; Liu T.; Yan L.; Liu J.; Dong F.; Ling M.; Liang C.; Lin Z. Metal-Organic Framework Nanosheets-Guided Uniform Lithium Deposition for Metallic Lithium Batteries. Energy Storage Mater. 2018, 11, 267–273. 10.1016/j.ensm.2017.11.003.

[ref45] Liu X.; Liu J.; Qian T.; Chen H.; Yan C. Novel Organophosphate-Derived Dual-Layered Interface Enabling Air-Stable and Dendrite-Free Lithium Metal Anode. Adv. Mater. 2020, 32, 1902724 10.1002/adma.201902724. 31777980

[ref46] Quilty C. D.; Bock D. C.; Yan S.; Takeuchi K. J.; Takeuchi E. S.; Marschilok A. C. Probing Sources of Capacity Fade in LiNi0.6Mn0.2Co0.2O2 (NMC622): An Operando XRD Study of Li/NMC622 Batteries during Extended Cycling. J. Phys. Chem. C 2020, 124, 8119–8128. 10.1021/acs.jpcc.0c00262.

