# Supplementary material for: Optimization of Electrode and Cell Design for Ultrafast-Charging Lithium-Ion Batteries Based on Molybdenum Niobium Oxide Anodes
Source: ACS Appl Energy Mater. 2022 Aug 12;5(9):11229–40. doi: 10.1021/acsaem.2c01814 (PMC9516618; doi:10.1021/acsaem.2c01814)
Supplement: Supplementary file 1 — ae2c01814_si_001.pdf [file ae2c01814_si_001.pdf]

Optimisation of electrode and cell design for ultrafast-charging lithium-ion batteries based on molybdenum niobium oxide anodes – **Supporting Information**

Yazid Lakhdar [a*], Harry Geary [b], Maurits Houck [b], Dominika Gastol [a] Alexander S. Groombridge [b], Peter R. Slater [c], Emma Kendrick [a]

[a] School of Metallurgy and Materials, University of Birmingham, Edgbaston Birmingham, B15 2TT, UK
[b] Echion Technologies Ltd, 9 Cambridge South, West Way, Sawston, Cambridge CB22 3FG, UK
[c] School of Chemistry, University of Birmingham, Edgbaston Birmingham, B15 2TT, UK

* Corresponding author: Yazid Lakhdar, y.lakhdar@bham.ac.uk

Cell Assembly

Table S.1 presents the half and full cell building details, including information on positive electrodes (PE), negative electrodes (NE), separators, and electrolyte volume (1M $LiPF_6$ in 50:50 EC:DEC v/v).

Table S.1: Half and full cells building information

Cell type	Cell geometry	Positive electrode	Negative electrode	PE surface area (cm^2)	NE surface area (cm^2)	Separator thickness (µm)	Electrolyte Volume (µL)
Cathode half	Coin	NMC622	Lithium	1.72	1.77	16	70
Anode half	Coin	MNO	Lithium	1.77	1.77	16	70
Full	Coin	NMC622	MNO	1.72	1.77	16	70
Cathode half	3-electrode	NMC622	Lithium	2.54	2.54	220	100
Anode half	3-electrode	MNO	Lithium	2.54	2.54	220	100
Full	3-electrode	NMC622	MNO	2.54	2.54	220	100
Full	Single-layer Pouch	NMC622	MNO	9	9.61	16	380

NMC622 electrode optimisation

NMC622/Li half-cells were manufactured in the dry room to validate the electrochemical behaviour and performance of the commercial cathode material to be used against $MoNb_{12}O_{33}$ in subsequent full cells. NMC622 was chosen as the cathode for subsequent full cells with the MNO anode as it is a well-known cathode material with good rate performance and good cycle life. Other cathode materials, such as NCA, could have been chosen. Nevertheless, NMC622 has a higher capacity than NMC111 and LFP, and it also has a longer cycle life than NMC811.

Figure S1.a shows the formation curve and its associated dQ/dV plot of three NMC622/Li half cells of various electrode densities. All cathodes were manufactured from the same starting electrode coating with an areal capacity of 0.95 ±0.05 mAh cm^{-2}. As the calendaring pressure was increased – i.e., as the porosity and thickness of the electrode was reduced – the reversible specific delithiation capacity at 10 mA g^{-1} could be gradually increased from 166 to 172 and then 179 mAh g^{-1} with cathode densities of 2.8, 3.2, and 3.4 g cm^{-3}, respectively, with a clear decrease in capacity fade as well. The coulombic efficiency of the cell with the highest porosity was 87.8% in the first cycle and 99.0% in the second cycle, while the electrode of highest density reached 90.5% and 99.0% coulombic efficiency in its first and second formation cycles, respectively.

The same asymmetric discharge rate tests as for the MNO/Li anode half cells were performed on the NMC622/Li cells. **Figure S1.b** displays the discharge (lithiation) capacity at these various rates, showing that the rate performance of these NMC622 cathodes in terms of capacity retention was not affected by calendaring, although higher values of capacity were obtained with decreasing electrode porosity and thickness.

Following rate testing, the same cells were then subjected to long-term cycling at 25°C. **Figure S1.c** demonstrates the excellent long term cyclability of NMC622/Li half cells cycled by applying a CCCV charge at 100 mA g^{-1} and a CC discharge at 200 mA g^{-1} until the discharge capacity fell to 70% of its initial value. The key SOH levels of 95, 90, 80, and 70% were reached after 120, 250, 650, and 800 cycles, respectively.

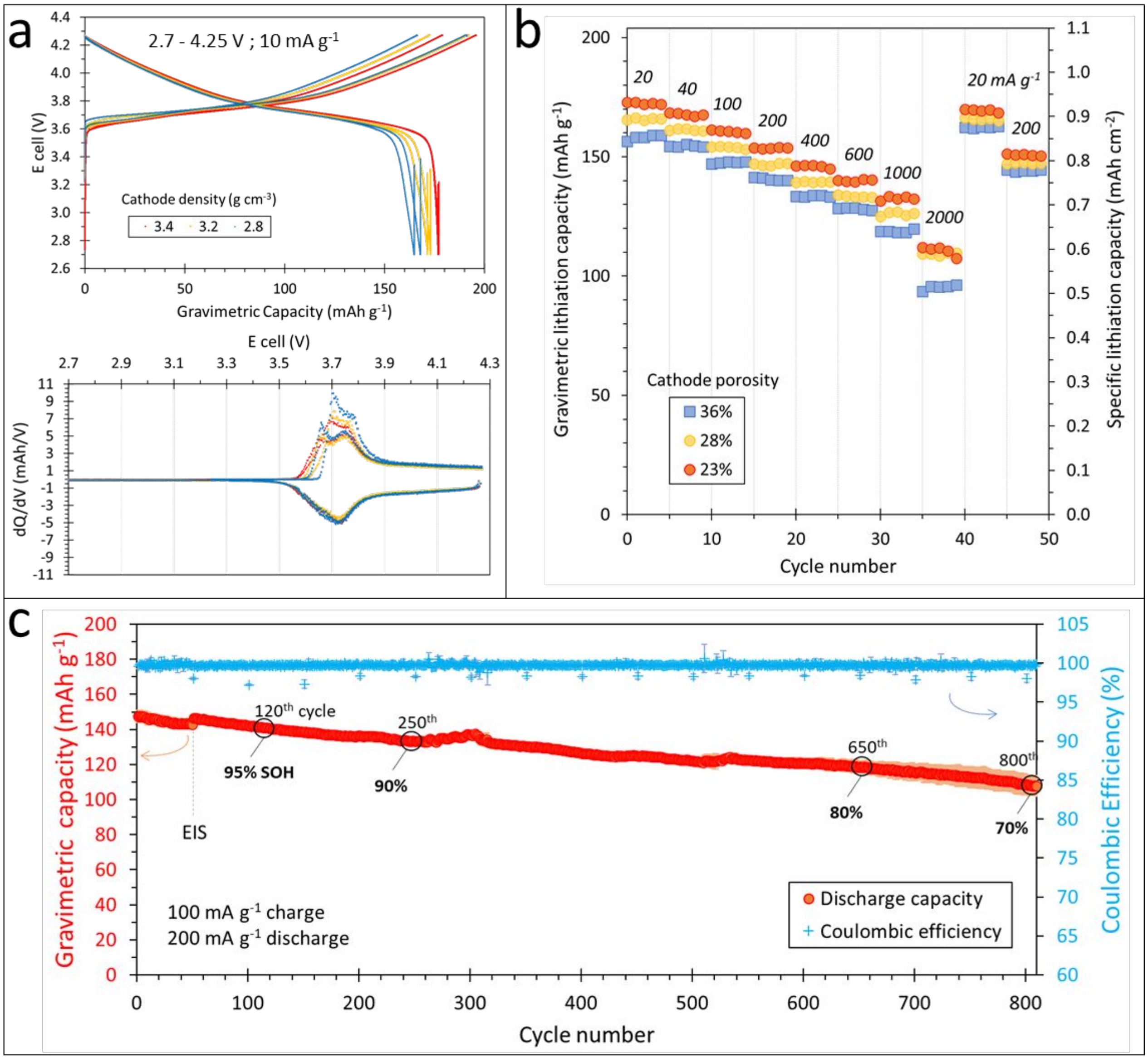

Figure S1: Galvanostatic cycling of NMC622/Li cathode half cells. Formation (a), asymmetric lithiation rate test (b) and long-term cycling at 25°C at 100/200 mA g-1 delithiation/lithiation(c).

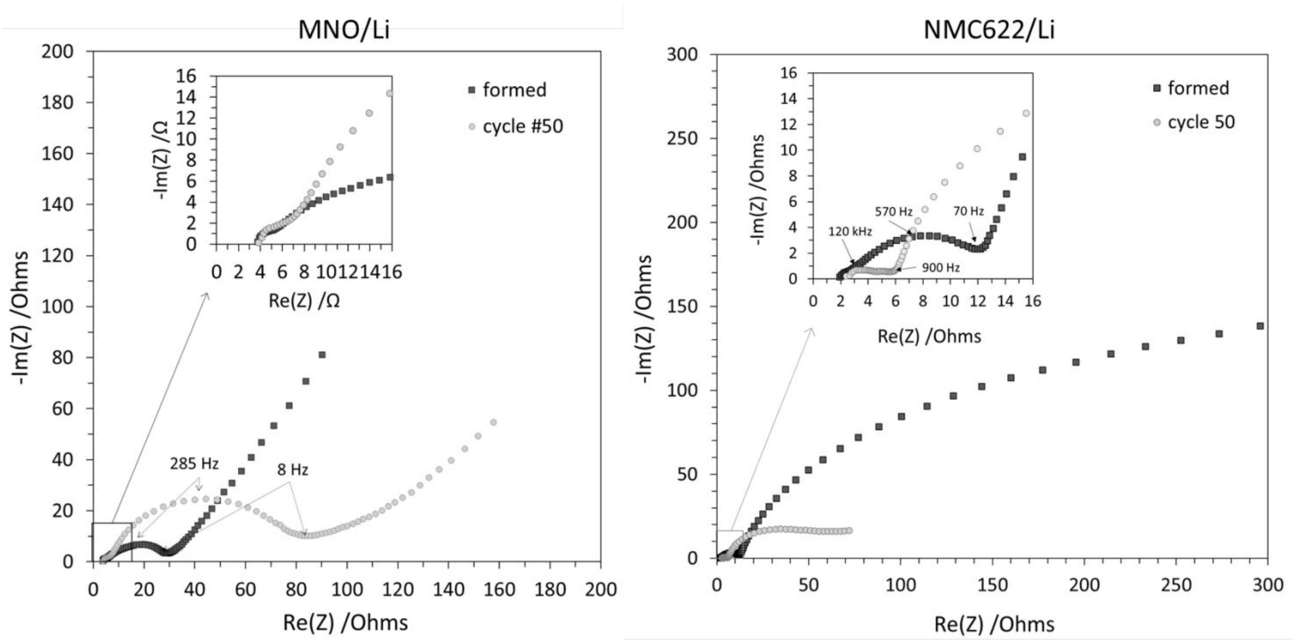

Figure S2: PEIS plots of the modified MoNb12O33/Li anode (a) and the NMC622/Li cathode (b) half cells after formation and after 50 cycles. PEIS was performed using a 5 mV amplitude in the frequency range 500 kHz – 10 mHz.

MNO/NMC622 full cell balancing

Figure S3 shows the galvanostatic charge-discharge formation curves and their associated dQ/dV graph of multiple MNO/NMC622 full-coin cells with N/P ratios of 0.9, 1.0, 1.1, and 1.2, cycled at 10 mA g^{-1} between 1.1 and 3.1 V.

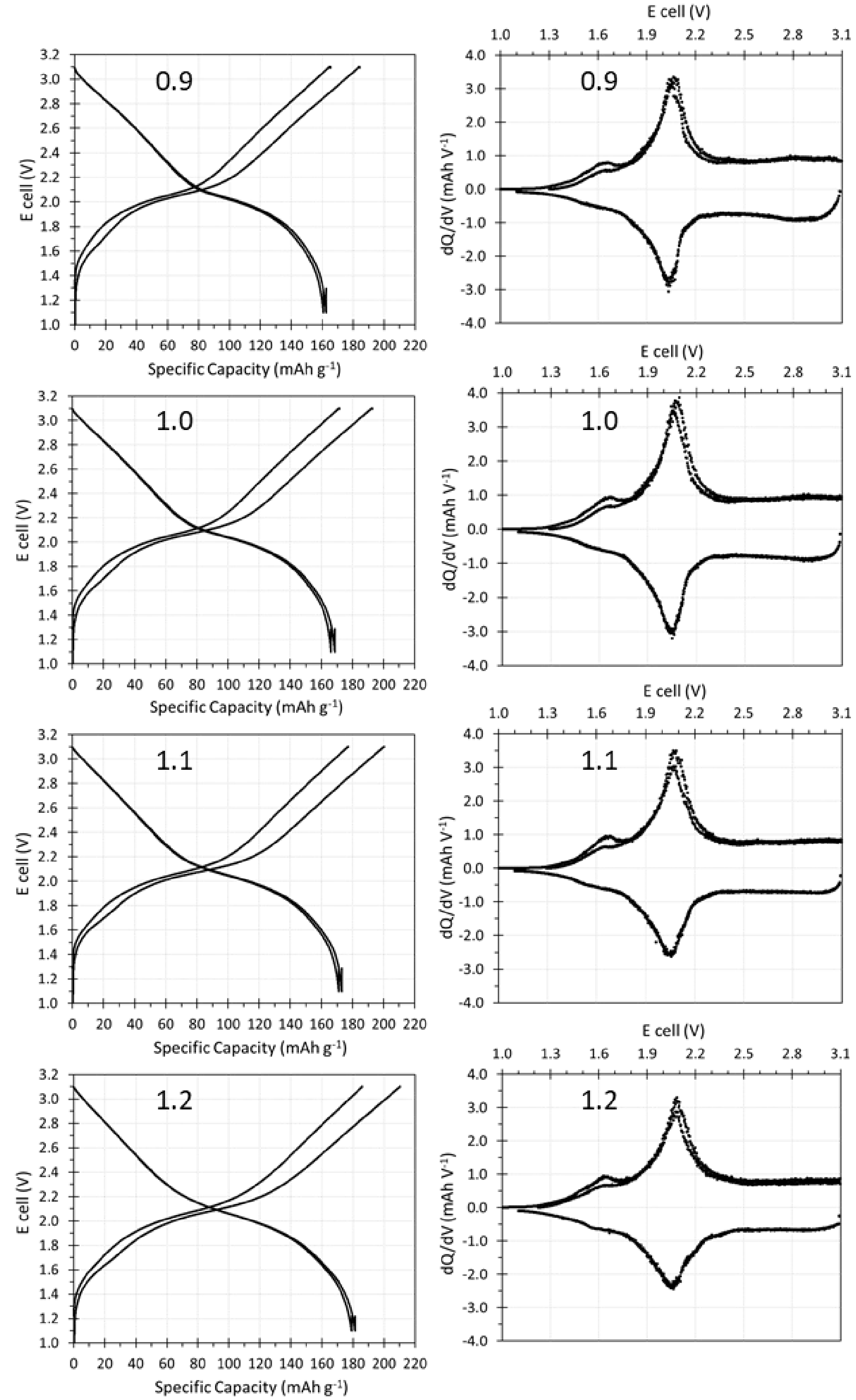

Figure S3: Formation profile and dQ/dV plots of MNO/NMC622 full coin cells with N/P capacity ratio varied from 0.9 to 1.2.
